# Application of nuclear magnetic resonance spectroscopy in food adulteration determination: the example of Sudan dye I in paprika powder

**DOI:** 10.1038/s41598-017-02921-8

**Published:** 2017-06-01

**Authors:** Yaxi Hu, Shuo Wang, Shenlin Wang, Xiaonan Lu

**Affiliations:** 1 Food, Nutrition and Health Program, Faculty of Land and Food Systems, The University of British Columbia, Vancouver, V6T 1Z4 BC Canada; 2 Key Laboratory of Food Nutrition and Safety, Ministry of Education of China, Tianjin University of Science and Technology, Tianjin, 300371 China; 3 Beijing Nuclear Magnetic Resonance Center, Peking University, Beijing, 100871 China; 4 College of Chemistry and Molecular Engineering, Peking University, Beijing, 100871 China; 5 Food Science Center of Peking University, Beijing, 100871 China

## Abstract

Carcinogenic Sudan I has been added illegally into spices for an apparent freshness. ^1^H solution and solid-state (SS) nuclear magnetic resonance (NMR) spectroscopies were applied and compared for determination of Sudan I in paprika powders (PPs). For solution NMR, PPs spiked with Sudan I were extracted with acetonitrile, centrifuged, rotor-evaporated, and re-dissolved in DMSO-d6 for spectral collection. For SSNMR, Sudan I contaminated PPs were mixed with DMSO-d6 solution and used for spectral collection. Linear regression models constructed for quantitative analyses resulted in the average accuracies for unknown samples as 98% and 105%, respectively. Limits of detection for the solution NMR and SSNMR spectrometers were 6.7 and 128.6 mg kg^−1^, while the limits of quantification were 22.5 and 313.7 mg kg^−1^. The overall analysis time required by both methods was similar (35 and 32 min). Both NMR techniques are feasible for rapid and accurate determination of Sudan I adulteration in PPs.

## Introduction

Food adulteration issues have been recorded ever since people started to process foods. With the increased complexity and globalization of food supply chain, the incidence rate of food fraud has increased extensively^1–3^. Among all types of food adulterations, deliberate addition of illegal chemicals (*e.g*. melamine in infant formula, formaldehyde in fruits, and Sudan I in paprika powder) into foods to reduce the cost or increase the gain could cause severe damage to consumers’ health. Therefore, accurate and rapid determination methods are required to identify food fraud and adulteration and thus guarantee the public health.

Conventional techniques to determine the existence of exotic chemicals in foods include chromatographic or immunological-based method coupled with various detection techniques^4–7^. Although these methods have high accuracy and sensitivity, they are generally time-consuming and labor-intensive because of complex sample pretreatment procedures, which impede the rapid and accurate determination of food fraud demanded by food industries and government laboratories. In addition, these methods were developed for the determination of specific chemical compound(s) whose appearance or unusual concentration demonstrates the adulteration. However, the deceptive behaviors are progressing dynamically. Organized criminals could develop novel adulteration methods adding chemical(s) that cannot be identified by the established methods. Consumers, government, and even food industries can be deceived by these appearing fraudulent products^8^. Rapid, accurate and non-targeted techniques are therefore appreciated in food industries and government laboratories to ensure the integrity of agri-food products.

Spectroscopic techniques [*i.e*. Raman spectroscopy, infrared (IR) spectroscopy, and nuclear magnetic resonance (NMR) spectroscopy] are the major fingerprinting techniques that collect signals from all chemicals present in a sample, thus can uncover any exotic materials that are not in the authenticated sample matrix, such as a food product. Raman and IR spectroscopies have been extensively investigated to reveal food fraud issues based upon their simple instrumentation, increased accessibility, and the rapid and non-destructive features^9–11^. However, these two spectroscopic techniques are not ideal to determine chemicals that are at low concentrations in food samples because of the low sensitivity. In addition, these two techniques respond to the vibration of functional groups in chemicals, but the variety of functional groups in chemicals is limited, overlapping signals of targeted molecules and interferents thus occur frequently. Moreover, functional groups behave differently in absorbing or scattering photons, making it not fully feasible to determine the absolute concentration of several target compounds using one reference chemical. On the contrary, NMR spectroscopy has a better performance to identify various chemicals because the minor difference in the electron cloud of nuclei can be accurately recorded by NMR spectra^12^. Thus, identification of chemicals sharing similar structure can be achieved^13, 14^. The intensity of NMR spectral signals are only influenced by the number of each type of nuclei present in the sample, thus absolute quantification of several specific chemicals simultaneously can be achieved by applying only one reference chemical^15, 16^. Although NMR spectroscopy also lack the sensitivity to determine the chemical(s) at low concentrations based on its principle, the sensitivity can be improved by simply increasing the number of scans during spectral collection without modifying any hardware.

NMR spectroscopy has become less expensive recently and its application has been generalized from analyzing the structure of pure biological samples^17, 18^ to the determination of chemicals in food products^19, 20^ and foodomics^21–24^. Most of the aforementioned studies were based upon solution NMR spectroscopy that requires liquefied samples, and thus extraction procedures are necessary for solid or semi-solid samples. In contrast, high-resolution magic-angle-spinning (HR-MAS) SSNMR spectroscopy could be an alternative to analyze samples in solid or semi-solid form directly. Quantitative analysis can be achieved by NMR spectroscopy utilizing several strategies, including internal standard, external standard, and linear regression^25^. A reference compound is required by both the internal and external standard strategies. Although with one reference compound, concentrations of several targeting compounds could be determined simultaneously, it could be troublesome to identify an appropriate reference compound under some circumstances (*e.g*. overlap of the signals of reference compound and sample matrices, interaction of the reference compound with sample matrices, etc.). Linear regression model correlates the concentration of target molecule with the integral of a specific NMR spectral peak (or a spectral region) and it could be applied to calculate this specific target molecule in new unknown samples.

To validate the feasibility of applying both solution NMR and SSNMR spectroscopies in the detection and quantification of hazard chemicals involved in food adulteration issues, the classic model of paprika powder contaminated with Sudan I was selected. Sudan I, a commonly used industrial dye for coloring clothes and wax, has been categorized as a third group genotoxic carcinogen by the International Agency for Research on Cancer^26^ and thus it is universally banned to be used as a food dye. However, Sudan I abuse in paprika powder and other culinary spice powders with the concentrations of 2.8–3500 mg kg^−1^ has been frequently reported since 2003 due to the economic gain that can provide a fake color of freshness^27^. Although numerous methods have been established for the determination of Sudan I in paprika powder, traditional methods including the chromatographic and immunological based methods are generally time-consuming and labor intensive. Therefore, the development of using NMR spectroscopic technique for rapid and accurate detection and quantification of Sudan I in paprika powder is of great significance. Because of the difficulty in finding appropriate reference compound, the potential error induced by reference sample preparation, and only one compound was to be analyzed in this study, linear regression method was adopted for quantitative analysis.

To the best of our knowledge, this was the first study to develop and validate NMR spectroscopic techniques for rapid and accurate detection and quantification of Sudan I in paprika powder (*i.e*. representative food adulteration by addition of illegal chemicals). Moreover, SSNMR spectroscopy was investigated unprecedentedly to determine Sudan I in paprika powder and its performance was compared with solution NMR spectroscopy.

## Results and Discussion

### Solvent selection

**Figure 1:**
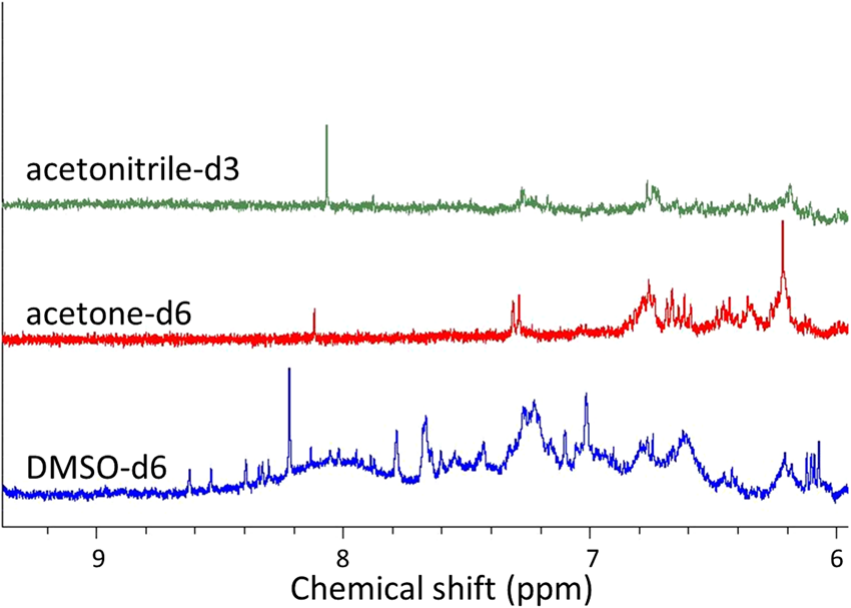
^1^H solution NMR spectra of paprika powder extracted by DMSO-d6 (bottom), acetone-d6 (middle), and acetonitrile-d3 (top).

To achieve the highest extraction efficiency of Sudan I from paprika powder and avoid simultaneous dissolving of impurities from paprika powder, an appropriate solvent with high solubility of Sudan I and low affinity towards compounds in paprika powder should be selected. ACN, DMSO, and acetone all performed high solubility of Sudan I, but their different polarity and molecular size could result in various affinities towards other chemicals. Thus, their affinities towards compounds in paprika powder were compared by ^1^H solution NMR spectrometer, and the results are shown in Fig. 1.

As the NMR resonances of Sudan I appear at the region of *ca*. 6.0–9.0 ppm, spectra at this specific region in Fig. 1 were compared. ACN had the least affinity towards compounds in paprika powder followed by acetone, while DMSO had significantly more peaks within this region, exhibiting the high solubility of paprika powder matrices in DMSO and low solubility in ACN. However, as heat can be generated using the high speed spinning in HR-MAS experiment, solvents with low boiling point might burst out of the rotor and cause damage to the instrument. Consequently, DMSO-d6 was selected as the solvent to extract Sudan I analyzed using HR MAS (boiling point of ACN, 82 °C; acetone, 56 °C; and DMSO, 182 °C). For solution NMR experiment, ACN was used to extract Sudan I from paprika powder, but DMSO-d6 was adopted to re-dissolve the products after vacuum evaporation, as different solvents result in shifts in peaks that are related to the target molecules and make the results of the two spectrometers difficult to compare.

### Determination of Sudan I by solution NMR spectroscopy

**Figure 2:**
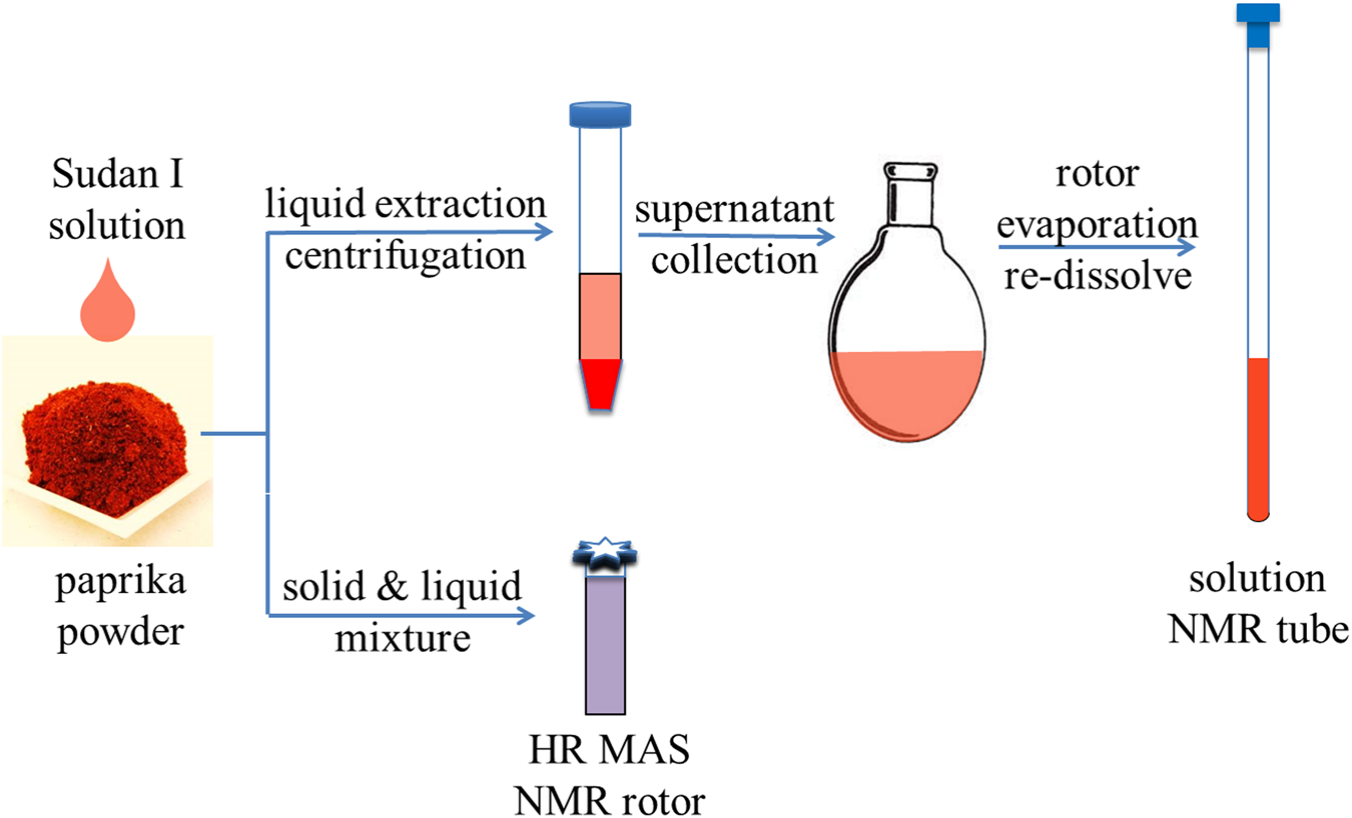
Schematic illustration of experimental design. NMR, nuclear magnetic resonance; HR MAS, high resolution magic angle spinning.

Paprika powder samples spiked with various Sudan I concentrations were determined using ^1^H solution NMR after extraction, vacuum evaporation, and re-dissolving, as demonstrated in Fig. 2.

**Figure 3:**
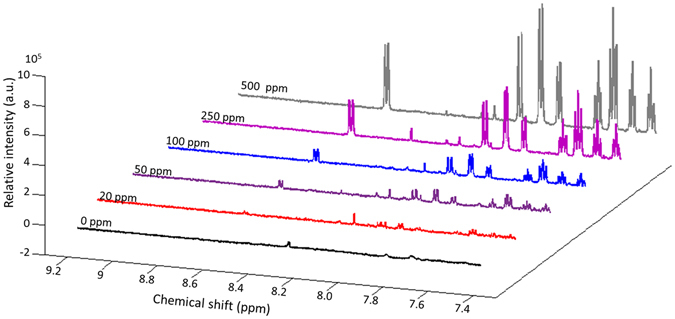
^1^H solution NMR spectra of paprika powder spiked with Sudan I at various concentrations (0, 20, 50, 100, 250, and 500 mg kg^−1^).

Paprika powder samples spiked with Sudan I at five different concentrations (*i.e*. 20, 50, 100, 250 and 500 mg kg^−1^) were analyzed using ^1^H solution NMR spectroscopy in triplicate on three consecutive days. Figure 3 shows the comparison of the spectra. The peaks at 8.57, 7.97, 7.88, 7.80, 7.62, 7.56, 7.47, 7.39, and 6.94 ppm are the nine featured Sudan I NMR peaks, denoting each proton in the Sudan I structure. To achieve a lower LOD and LOQ, the integral of the strongest peak at 7.88 ppm was used to quantify Sudan I in paprika powder.

The recovery of this proposed analysis was determined by comparing the spectra of the extraction from paprika powder sample spiked with Sudan I at 100 mg kg^−1^ (*i.e*. 40 mg L^−1^ Sudan I in the final extraction if 100% recovery is achieved) with the standard Sudan I DMSO-d6 solution at the same concentration. The identical intensities of these two spectra (Figure S1) validated the full recovery of Sudan I from paprika powder by the current sample pretreatment procedure. The RSD values of the triplicate samples at five different concentrations were in the range of 0.3–7.5%, with an average of 4.6%. The higher the concentration of Sudan I in paprika powder, the lower the RSD value. The low RSD values represent a good repeatability of this proposed ^1^H solution NMR spectroscopic method.

**Figure 4:**
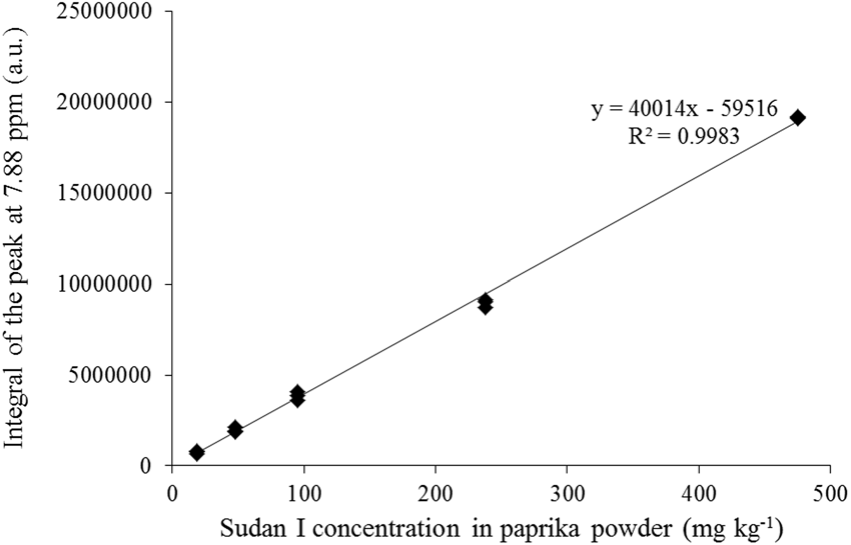
Linear regression model for quantitative analysis of Sudan I in paprika powder by ^1^H solution NMR spectrometer.

The linear regression model correlating the integrals of the peak at 7.88 ppm and the concentration of Sudan I in paprika powder is shown in Fig. 4. The high R^2^ (close to 1) and relatively small intercept of the regression equation represent high correlation and low baseline effect of the spectra at 7.88 ppm.

Five paprika powder samples spiked with Sudan I at different concentrations were prepared separately and treated as unknown samples for the determination of the accuracy of this proposed method. The linear regression model (Fig. 4) was applied to calculate the concentrations of Sudan I in the unknown samples, and the accuracy was in the range of 94–104% with an average of 98%. The high accuracy demonstrates the feasibility of accurate quantification of Sudan I in paprika powder using ^1^H solution NMR spectroscopy.

The LOD and LOQ were calculated based upon the IUPAC standard method^28^. The integrals of the spectra for the uncontaminated paprika powder samples were determined and the standard deviation was calculated accordingly. The LOD of this proposed method was 6.7 mg kg^−1^, while the LOQ was 22.5 mg kg^−1^. Both the LOD and the LOQ are within the range of commercial Sudan I contamination levels (*i.e*. 2.8–3500 mg kg^−1^).

Because NMR signals respond to the mole number of the chemicals present in the sample, specifically protons, the LOD and LOQ of this solution NMR spectroscopic method under the specific instrumentation are 5.5 and 18.1 nmol, respectively. By increasing the weight of paprika powder for extraction, the mole number of Sudan I contained in the final DMSO solution could be increased accordingly, leading to improved LOD and LOQ reported based upon the weight of paprika powder. Moreover, with an increased number of scans, the intensity of the signals as well as the signal-to-noise ratio could be improved, resulting in higher accuracy for the quantification of low concentration adulteration as well as lower LOD and LOQ.

### Determination of Sudan I by SSNMR spectroscopy

In general, sample pretreatment in HR-MAS SSNMR experiment is much simpler than our solution NMR spectroscopic method, resulting in significantly reduced labor (Fig. 2). To prepare the spiked samples, 10 mg paprika powder was packed into SSNMR rotor and mixed with 45 µl Sudan I DMSO-d6 solution, followed by HR-MAS NMR spectral collection.

The volume of the 4-mm rotor is 50 µl, which is about 10 times smaller than the volume of the sample analyzed by solution NMR spectroscopy. To achieve a similar signal-to-noise ratio, the number of scans should be set higher than the one used in solution NMR spectroscopy. Thus, spectral collection using SSNMR requires longer time than solution NMR spectroscopic method.

**Figure 5:**
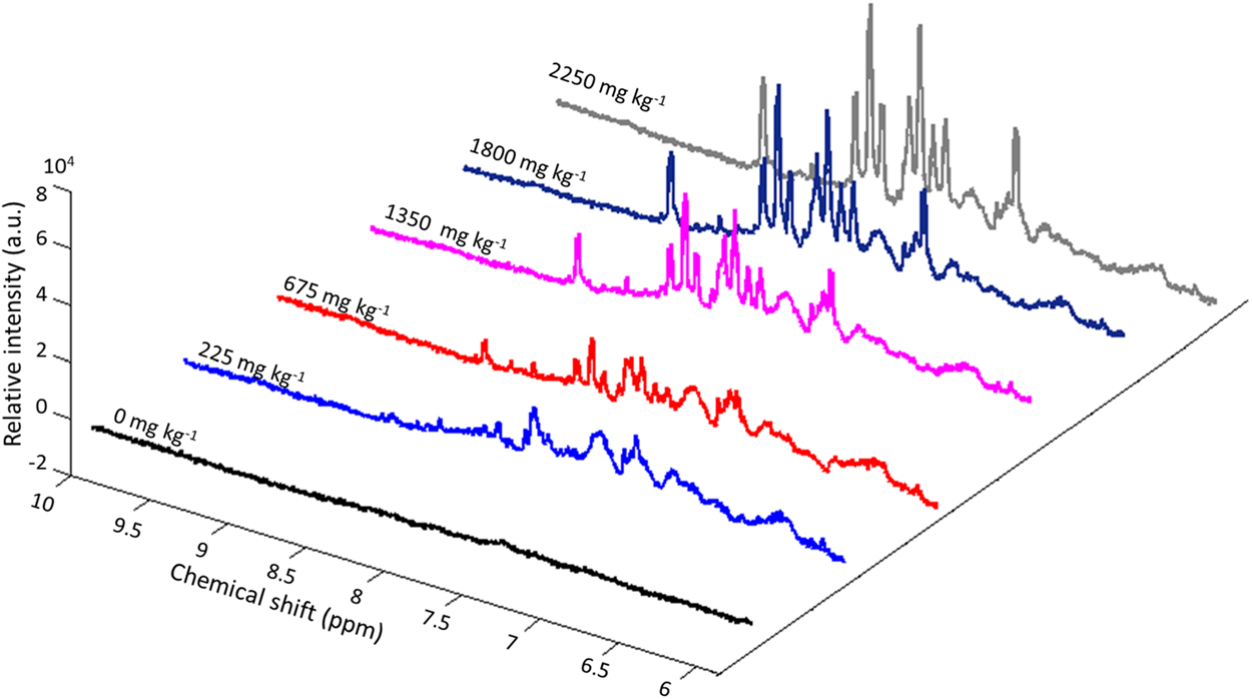
^1^H HR MAS SSNMR spectra of paprika powder spiked with Sudan I at various concentrations (0, 225, 675, 1350, 1800, and 2250 mg kg^−1^).

Triplicate of five standard paprika samples with various Sudan I concentrations (*i.e*. 225, 675, 1350, 1800, and 2250 mg kg^−1^) were analyzed using HR-MAS SSNMR spectroscopy in three consecutive days. Figure 5 exhibits the comparison of representative spectra of samples at five different concentrations. More bands were observed in Fig. 5 compared to Fig. 3 because of the high affinity of DMSO towards chemicals in paprika powder. However, no signal overlapping was observed at 7.88 ppm on the spectra and thus, the integral of the peak at 7.88 ppm was again used to quantify Sudan I in paprika powder.

RSD of triplicate samples at each concentration was calculated and were within the range of 1.5–6.7%, with an average of 3.9%. The low RSD values demonstrate a good repeatability of this proposed HR MAS SSNMR spectroscopic method.

**Figure 6:**
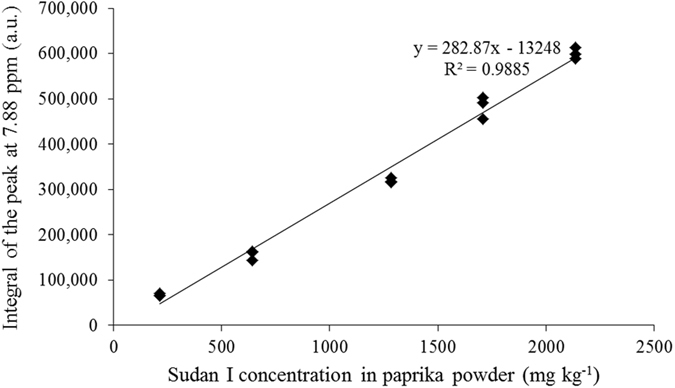
Linear regression model for quantitative analysis of Sudan I in paprika powder by ^1^H HR MAS SSNMR spectrometer.

The linear regression model constructed by five standard samples (*i.e*. 225, 675, 1350, 1800, and 2250 mg kg^−1^ Sudan I in paprika powder) in triplicate was constructed, and the results are shown in Fig. 6. The high R^2^ represents a good linear regression relationship between the concentration of Sudan I in paprika powder and the integral at 7.88 ppm in the spectra, which can be used to accurately quantify the concentration of Sudan I in paprika powder. However, the high intercept showed the interference of the spectral baseline or the compounds from paprika powder dissolved by DMSO, which could be optimized by using other solvents.

The accuracy was also determined by five unknown samples spiked with Sudan I at 337.5, 450, 1125, 1575, and 3150 mg kg^−1^, respectively. The accuracy values were within the range of 98–109% with an average of 105%. The accuracy of SSNMR spectroscopic method was not as good as the accuracy of the solution NMR spectroscopic method, which could be attributed to the small weight of the samples that are analyzed, leading to more experimental errors. The accuracy of this proposed SSNMR spectroscopic method could be improved by using NMR rotor with larger inner diameter, thus having larger volume to contain more paprika powder samples to be analyzed.

The LOD and LOQ are 128.6 and 313.7 mg kg^−1^, respectively, for the SSNMR spectroscopic method. Although higher than the values determined using ^1^H solution NMR spectroscopy, the LOD and LOQ determined using this proposed SSNMR spectroscopic method are still within the commercial Sudan I contamination level (*i.e*. 2.8–3500 mg kg^−1^), and can be further improved with the increased number of scans.

Reported in mole number, the LOD and LOQ of this HR-MAS SSNMR spectroscopic method under the given instrumentation are 5.2 and 17.3 nmol, respectively, which are similar to the ones determined using solution NMR spectroscopy. By using a rotor with larger diameter, the weight of the paprika powder samples analyzed using SSNMR spectroscopy could be increased, leading to increased mole number of Sudan I present in NMR spectrometer and subsequently reduced LOD and LOQ reported based upon the weight of paprika powder.

The overall analysis time required for solution NMR and SSNMR spectroscopies were 35 min and 32 min, respectively. Although SSNMR spectroscopy exhibited relatively lower sensitivity compared to solution NMR spectroscopy based upon the LOD and LOQ values reported on the weight of paprika powder, substantial time and labor can be saved by using SSNMR spectroscopy when a large sample set is to be analyzed (*e.g*. 60 min could be saved to analyze 20 samples).

### Comparison of techniques for the determination of Sudan I in food samples

Extensive studies have been conducted to improve and optimize the determination of Sudan I in paprika powder, such as chromatographic-based ultraviolet (UV) spectroscopic or mass spectrometric (MS) methods^6, 26, 29–31^, immunological-based colorimetric methods^32–34^, as well as Raman and infrared spectroscopic methods^35–38^.

Amongst these aforementioned techniques, immunological-based colorimetric methods exhibited high sensitivity for the detection of spice contaminated with Sudan I at several part per billions levels^32^. However, these methods are generally time-consuming, requiring several hours to complete the labor-intensive sample pretreatment and analytical procedures^33^. A modified immunological-based colorimetric method, reported by Oplatowska and others^34^, can complete the overall immunological assay for the detection of Sudan I in spice power in *ca*. 20 min, but the high RSD demonstrated the poor repeatability and accuracy for quantitative analysis. Moreover, the production of appropriate antibody is generally time consuming and expensive, and strict environmental conditions are demanded for the storage and application of immunological-based methods, posing challenges if these methods are to be adopted by food industries or government laboratories for the analysis of large sample sets.

Chromatographic-based UV or MS methods have comparable or even higher sensitivity than immunological assays, and exhibited the highest accuracy amongst these techniques^29, 31^. However, these chromatographic based methods are also time-consuming and labor intensive^6, 30^, and do not address the rapid analysis required by food industries and government laboratories. In addition, filtration procedures, commonly used to remove macromolecules and undissolved food matrices that might interfere with the chromatographic analysis or cause damage to the instrument^29, 30^, could potentially induce a loss of Sudan I and thus the underestimation of the concentration of Sudan I due to the adsorption by the filter (Figure S2).

Raman and infrared spectroscopic methods are the most rapid ones. According to Haughey and others^36^, no sample pretreatment is required to analyze the Sudan I contaminated chili powder, and the spectral collection only took a few minutes. Moreover, the linear regression models demonstrated high potential for accurate quantification of Sudan I in chili powder samples. However, the LOD for the near-infrared and Raman spectroscopies were 2500 and 8800 mg kg^−1^, respectively, based on the weight of chili powder, which are too high to be used by food industry and government laboratory. Surface enhanced Raman spectroscopy (SERS), a derivative of the conventional Raman spectroscopy, has been applied for the determination of Sudan I in chili powder to reduce the LOD to 0.6 mg kg^−1^ with the assistance of well-designed noble metallic nanostructures^37^. However, extensive sample pretreatment is required to remove the majority of food components (*i.e*. liquid-liquid extraction combined with solid phase extraction) because the SERS substrate non-selectively enhanced the Raman scattering signals of all organic matters present in a sample^39^. With an added separation element, such as molecularly imprinted polymers, more efficient extraction and separation of Sudan I can be achieved and the analysis time has been reduced to less than 15 min^35^; however, the identification of the enhanced Raman signals for Sudan I might be problematic and the reproducibility of the SERS spectra are not ideal.

Compared to the other well-established methods, NMR spectroscopic methods are still in their infancy for the determination of chemical hazards in foods. ^1^H solution NMR spectroscopy has been applied to differentiate four types of Sudan dyes in spice powders at high concentrations^14, 40^, but quantification of Sudan I in food samples was not conducted in any previous study. Our current study is also the first time that SSNMR spectroscopy has been applied for the detection and quantification of Sudan I in food matrices. In general, both of these two spectroscopic methods exhibited extraordinary performance in their repeatability, with RSD values similar to those for chromatographic-based methods^31^. The high R^2^ and accuracies for the quantification of unknown samples validated the outstanding quantification capability. In addition, simple sample pretreatment for NMR spectroscopies results in improved recovery of the target molecules during analysis and substantial time and labor have been saved (*i.e*. 35 min for solution NMR spectroscopy and 32 min for SSNMR spectroscopy). The weaknesses of NMR spectroscopic techniques are the relative high instrumental costs and the relatively low sensitivity for the detection of trace level compounds. Because of the simple and rapid sample pretreatment and analysis, the costs for the analysis of each sample using NMR spectroscopy are affordable; with increased amount of samples extracted, increased probe size of SSNMR spectrometer or increased number of scans, an improved sensitivity could be achieved. It is worth mentioning that more than 100 mg kg^−1^ Sudan I is required to provide a change in the color freshness of paprika powder^31^, making these NMR spectroscopic methods adequate to identify the intentional addition of Sudan I into paprika powder for commercial benefits. Finally, as a fingerprinting technique analogous to infrared and Raman spectroscopies, NMR spectroscopy can be applied as a non-targeted method to identify exotic materials present in the food system and thus reveal novel types of food adulteration and fraud issues.

Although the price for NMR spectrometers are relatively high (*c.a*. three to four times the price as for Raman spectrometer), the cost for the analysis of each sample is affordable (*c.a*. several to dozens of Canadian dollars). With the advance in the magnetic materials and instrumental hardware, the cost for conventional NMR spectrometers could be reduced. In addition, benchtop NMR spectrometer could have improved sensitivity to be applied for the determination of chemicals at low concentrations. Further studies could be conducted to develop one simple method for the simultaneous detection and quantification of various chemical hazards present in food matrices to substitute the comprehensive multiple tests, resulting in less labor, time and money involved.

## Materials and Methods

### Materials

Sudan I (≥95% purity) was purchased from Sigma Aldrich (Oakville, Ontario). Reagent grade acetonitrile (ACN) and dimethyl sulfoxide (DMSO) was acquired from Fisher Scientific (Toronto, ON). Deuterated solvents including DMSO-d6, ACN-d3, and acetone-d6 were obtained from Cambridge Isotope Laboratories (Tewksbury, MA). Three bags of paprika powder samples of the same brand were purchased from local grocery stores in Vancouver, Canada. Sudan I stock solution at 2500 mg L^−1^ in ACN and DMSO were prepared using 50 mL volumetric flasks.

### Solvent optimization

To achieve high extraction efficiency towards Sudan I and low affinity to compounds in paprika powder matrix, the solvent to be used to extract Sudan I from paprika powder was optimized. Three solvents (*i.e*. ACN-d3, acetone-d6, and DMSO-d6) with high solubility of Sudan I were evaluated. Briefly, 250 mg of unadulterated paprika powder was mixed with 500 µL of the three selected solvents, respectively. After centrifugation at 4500 × *g* for 5 min, 400 µL of the supernatant was transferred to a standard 5-mm solution NMR tube for spectral collection. The spectra were compared between solvents, and the solvent with the fewest peaks in the Sudan I region of the NMR spectrum (*i.e*. 6.0–9.0 ppm) was selected as the optimum extraction solvent.

### Sample preparation

Paprika powder samples were determined to be free from Sudan I by high performance liquid chromatography-photodiode array detector (HPLC-DAD) following the same procedures as Fang and coauthors^35^. Sudan I stock solution in ACN was diluted to various concentrations with ACN, while the stock solution in DMSO was diluted with DMSO-d6 to reach a final solution containing 50% DMSO-d6.

To prepare the samples analyzed by solution NMR, Sudan I was extracted from paprika powder following a procedure similar to that for HPLC-DAD analysis with small modifications. Briefly, 200 mg of paprika powder samples were spiked with 400 µL of Sudan I ACN solutions at various concentrations. After adding 4 mL ACN, the adulterated samples were vortexed for 30 s, sonicated for 10 min, and centrifuged at 4500 × *g* for 5 min. Each supernatant was collected and vacuum evaporated to dryness, followed by re-dissolved in 500 µL DMSO-d6. An aliquot of 450 µL of the extracted samples were loaded into standard 5-mm NMR tubes. Paprika powder samples spiked with Sudan I at 20, 50, 100, 250, and 500 mg kg^−1^ were prepared three times on three consecutive days for the determination of the linearity and repeatability. To evaluate the accuracy, paprika powder samples spiked with 50, 75, 125, 200, and 800 mg kg^−1^ Sudan I were prepared following the same procedures and regarded as unknown samples.

For HR-MAS SSNMR spectroscopy, 10 mg samples of paprika powder were transferred directly into standard 4 mm HR-MAS rotors, spiked with 45 µL Sudan I in 50% DMSO-d6 at a series concentration, and capped tightly with insert and rotor cap, followed by spectral collection. Paprika powder samples spiked with Sudan I at 225, 675, 1350, 1800, and 2250 mg kg^−1^ were prepared three times in three consecutive days to determine the linearity and repeatability. To evaluate the accuracy, paprika powder samples with 337.5, 450, 1125, 1575, and 3150 mg kg^−1^ Sudan I were prepared following the same procedures and treated as unknown samples.

### Instrumentation

^1^H solution NMR spectroscopic experiments were conducted using a Bruker’s spectrometer operating at proton frequency of 600.13 MHz equipped with a TXI probe. The sample temperatures were controlled at 300.0 K. All spectra were collected using a single pulse with a 30° pulse angle, 2.65 s acquisition time, 4 s recycle delay, and a sweep width of 20. ppm. The number of scans was 128, requiring *ca*. 15 min for each spectral collection.

^1^H HR-MAS SSNMR spectroscopic experiments were performed using a wide-bore Bruker spectrometer operating at proton frequency of 600.25 MHz equipped with a 4.0 mm 1 H/X/Y probe. The sample temperatures were controlled at 300.0 K and the samples were spun at 7 kHz. The spectra were collected using a pulse sequence with a 90° pulse angle, 1 s acquisition time, 4 s recycle delay, and a sweep width of 20.02 ppm. The solvent signal was suppressed by pre-saturation at the frequency of DMSO resonance. The number of scans for the experiments was set at 384, with total experimental time of *ca*. 32 min per sample.

### Spectral analysis

TopSpin 3.2 software (Bruker Biospin GmbH, Rheinstetten, Germany) was applied to preprocess all NMR spectra that were collected using both solution NMR and SSNMR spectrometers. Spectra were phased and baseline corrected manually. After peak assignments, integration was applied to analyze the resonances of Sudan I.

The recovery of Sudan I from paprika powder for solution NMR analysis was evaluated by comparing the spectral features of the extracts from contaminated paprika powder samples with the standard Sudan I solution at the same concentration. Assessment of recovery was not required for SSNMR analysis because no sample pretreatment was involved to cause the loss of target chemical. The relative standard deviation (RSD) was calculated based upon Equation S1 to evaluate the repeatability of the experimental design. Linear regression models correlating Sudan I concentrations and the integrals of the peak were constructed using Microsoft Excel 2010 (Redmond, WA) and the linearity was represented by the coefficient of determination (R^2^). The concentrations of the unknown samples were calculated using the established linear regression models, and the accuracy was evaluated using Equation S2. The limit of detection (LOD) and limit of quantification (LOQ) were determined as three times the standard deviation of noise and 10 times the standard deviation of noise^28^, respectively.

### Data availability

The datasets generated during and analyzed during the current study are available from the corresponding author on reasonable request.

## Conclusion

Both the solution NMR and SSNMR spectroscopies can be used to rapidly and accurately quantify Sudan I in paprika powder. The average accuracy for the quantification of Sudan I in unknown samples using solution NMR spectroscopy was 98%, while the accuracy of SSNMR spectroscopy was 105%. Based upon the weight of paprika powder, the LOD and LOQ were 6.7 and 22.5 mg kg^−1^ for solution NMR spectroscopy, and 128.6 and 313.7 mg kg^−1^ for SSNMR spectroscopy. The LOD and LOQ were 5.5 and 18.1 nmol for solution NMR spectroscopy and 5.2 and 17.3 nmol for SSNMR spectroscopy, if calculated based upon the mole number of Sudan I in NMR spectrometers. LOD, LOQ, and accuracy can be further improved by increasing either the weight of paprika powder or number of scans. The overall analysis time was 35 min and 32 min, respectively, reducing half of the analysis time required by traditional analyses. As a fingerprinting technique, NMR could identify any exotic chemicals in foods to reveal novel types of adulteration, and thus assure the integrity and safety of food system.

## Electronic supplementary material

Supplementary information
