# Supplementary material for: Application of nuclear magnetic resonance spectroscopy in food adulteration determination: the example of Sudan dye I in paprika powder
Source: Sci Rep. 2017 Jun 1;7:2637. doi: 10.1038/s41598-017-02921-8 (PMC5454000; doi:10.1038/s41598-017-02921-8)
Supplement: Supplementary file 1 — Supplementary information [file 41598_2017_2921_MOESM1_ESM.pdf]

Application of nuclear magnetic resonance spectroscopy in food adulteration determination: the example of Sudan dye I in paprika powder

Yaxi Hu[1], Shuo Wang[2], Shenlin Wang[3,4,5*], Xiaonan Lu[1*]

[1]Food, Nutrition and Health Program, Faculty of Land and Food Systems, The University of British Columbia, Vancouver, V6T 1Z4, BC, Canada

[2]Key Laboratory of Food Nutrition and Safety, Ministry of Education of China, Tianjin University of Science and Technology, Tianjin, 300371, China

[3]Beijing Nuclear Magnetic Resonance Center, Peking University, Beijing, 100871, China

[4]College of Chemistry and Molecular Engineering, Peking University, Beijing, 100871, China

[5]Food Science Center of Peking University, Beijing, 100871, China

Equations

$$\mathrm{RSD} = (standard\ deviation/mean) \times 100 \qquad \text{(S1)}$$

$$\mathrm{Accuracy} = (calculated\ concentration/spiked\ concentration) \times 100 \qquad \text{(S2)}$$

Figures

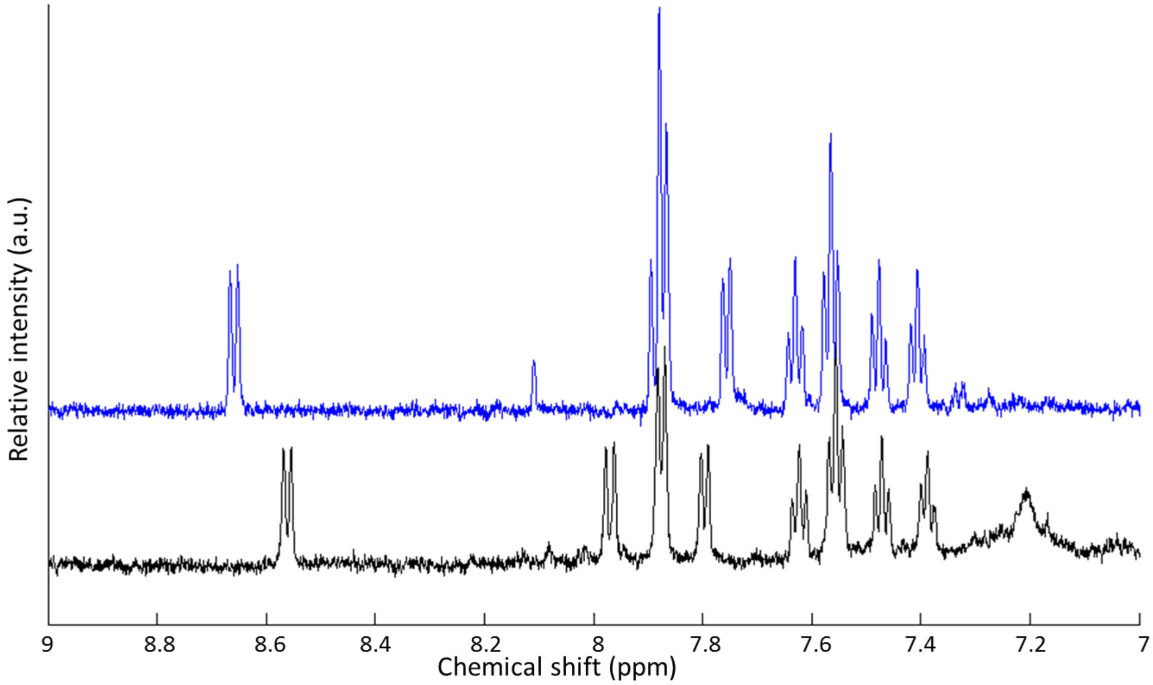

Figure S1. ^{1}H solution NMR spectra of paprika powder spiked with Sudan I at 100 mg kg^{-1} (bottom, black) and standard Sudan I ACN solution at 40 mg L^{-1} (top, blue). The shift of the peaks was because of the different solvent used for these two spectra.

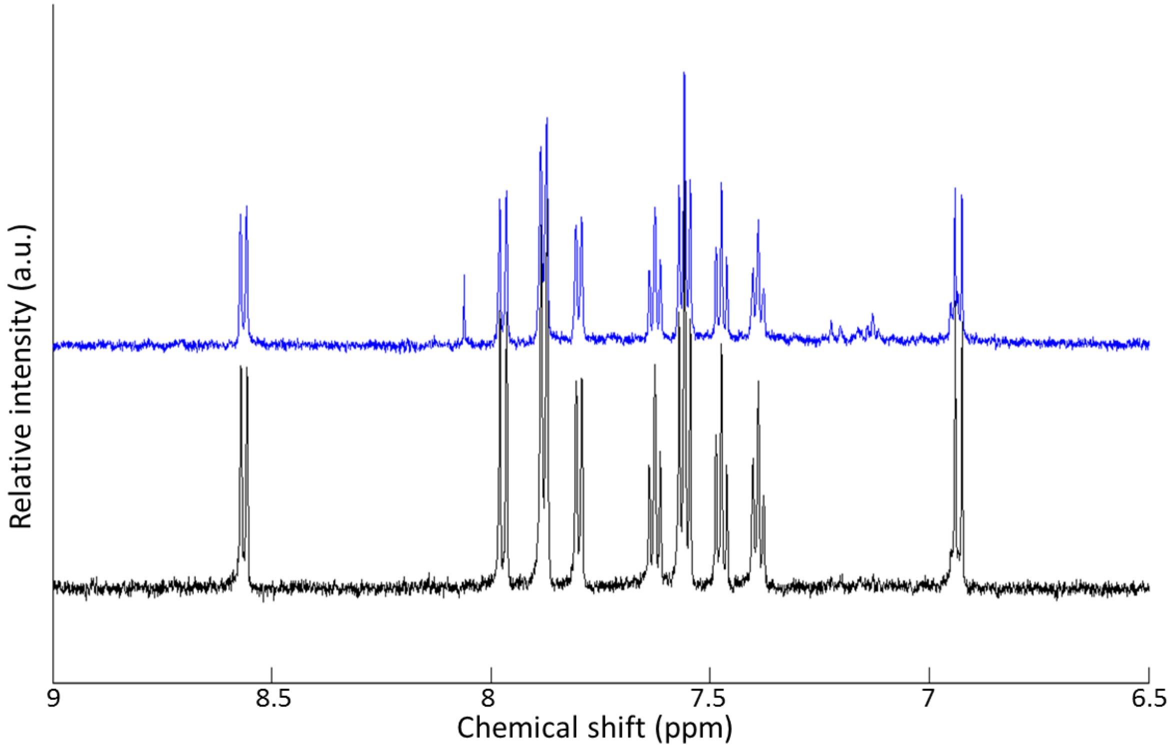

Figure S2. ^{1}H solution NMR spectra of 100 mg L^{-1} Sudan I standard solution in ACN without being filtered (bottom, black) and 100 mg L^{-1} Sudan I standard solution in ACN after being filtered by 0.45 μm nylon syringe filter (top, blue). Approximately 40% Sudan I lost during the filtration.
